# Prognostic Signature for Lung Adenocarcinoma Patients Based on Cell-Cycle-Related Genes

**DOI:** 10.3389/fcell.2021.655950

**Published:** 2021-03-18

**Authors:** Wei Jiang, Jiameng Xu, Zirui Liao, Guangbin Li, Chengpeng Zhang, Yu Feng

**Affiliations:** ^1^Department of Thoracic Surgery, The First Affiliated Hospital of Soochow University, Suzhou, China; ^2^Department of Neurology, The Second Affiliated Hospital of Soochow University, Suzhou, China; ^3^Medical College, Orthopedic Institute, Soochow University, Suzhou, China

**Keywords:** lung adenocarcinoma, cell cycle-related genes, prognostic signature, overall survival, GEO

## Abstract

**Objective:**

To screen lung adenocarcinoma (LUAC)-specific cell-cycle-related genes (CCRGs) and develop a prognostic signature for patients with LUAC.

**Methods:**

The GSE68465, GSE42127, and GSE30219 data sets were downloaded from the GEO database. Single-sample gene set enrichment analysis was used to calculate the cell cycle enrichment of each sample in GSE68465 to identify CCRGs in LUAC. The differential CCRGs compared with LUAC data from The Cancer Genome Atlas were determined. The genetic data from GSE68465 were divided into an internal training group and a test group at a ratio of 1:1, and GSE42127 and GSE30219 were defined as external test groups. In addition, we combined LASSO (least absolute shrinkage and selection operator) and Cox regression analysis with the clinical information of the internal training group to construct a CCRG risk scoring model. Samples were divided into high- and low-risk groups according to the resulting risk values, and internal and external test sets were used to prove the validity of the signature. A nomogram evaluation model was used to predict prognosis. The CPTAC and HPA databases were chosen to verify the protein expression of CCRGs.

**Results:**

We identified 10 LUAC-specific CCRGs (PKMYT1, ETF1, ECT2, BUB1B, RECQL4, TFRC, COCH, TUBB2B, PITX1, and CDC6) and constructed a model using the internal training group. Based on this model, LUAC patients were divided into high- and low-risk groups for further validation. Time-dependent receiver operating characteristic and Cox regression analyses suggested that the signature could precisely predict the prognosis of LUAC patients. Results obtained with CPTAC, HPA, and IHC supported significant dysregulation of these CCRGs in LUAC tissues.

**Conclusion:**

This prognostic prediction signature based on CCRGs could help to evaluate the prognosis of LUAC patients. The 10 LUAC-specific CCRGs could be used as prognostic markers of LUAC.

## Introduction

Lung cancer (LC) remains one of the most common malignancies and is a major contributor to cancer-related deaths worldwide, accounting for 11.6% of cancers and 18.4% of deaths, respectively (Bray et al., 2018). In China, the burden of LC remains the highest of all cancers, with mortality and incidence rates 1.5 times those worldwide in 2017 (Liu et al., 2020). Non-small-cell LC (NSCLC) is the predominant type (approximately 85%) of LC; it includes lung adenocarcinoma (LUAC) and lung squamous cell carcinoma (LUSC), of which LUAC is the most prevalent type (Peng et al., 2017; Herbst et al., 2018). LUAC originates in the distal airway and has less correlation with chronic inflammation and smoking than LUSC (Peng et al., 2017).

Currently, anatomical surgical resection and mediastinal lymph node dissection are the most effective methods for treatment of early stage LUAC patients, and the main surgical procedures are lobectomy and sub-lobectomy (Lin et al., 2020; Yu et al., 2020). Advanced-stage LUAC covers a variety of disease manifestations and has an equally complex range of multimodal treatment options, including systemic and local therapies (chemotherapy, radiation therapy, etc.) for remote and local symptom control, respectively (Evison, 2020). With the application of biomarker-directed therapies targeting molecular changes (such as EGFR and BRAF V600E mutations, or ALK and ROS1 rearrangements), these therapies can prolong the survival of LC patients (Arbour and Riely, 2019). In the past few years, with the application of high-throughput sequencing (Illumina HiSeq, Illumina MiSeq, Ion PGM^TM^, etc.), increasing numbers of rare molecular changes in oncogenic drivers (including HER2, MET, and RET) have been identified (Yu et al., 2018). Specific tyrosine kinase inhibitors targeting these genomic changes have shown improved patient survival and satisfactory biological activity, mostly in phase III clinical trials, which has led the US Food and Drug Administration to accelerate the approval of some of these drugs (Lamberti et al., 2020). However, the 5 year survival rate has only increased by 5% in the past 20 years (Johnson et al., 2014).

Increasing numbers of studies show that the cell cycle is tightly bound to the growth and proliferation of LUAC cells, with certain genes potentially functioning as cycle regulators. For example, knockdown of GINS2 induced cell cycle arrest and apoptosis in A549 cells (Sun et al., 2021), and MITF could inhibit NSCLC progression by controlling the cell cycle (Hsiao et al., 2020). However, the results of such studies are difficult to translate into clinical practice. This is mainly because tumor occurrence and development are pathological processes driven by multiple genes and cannot adequately be explained by the abnormal expression of a single gene. Although changes in a certain gene may lead to differences in the prognosis of patients, the sensitivity and specificity of clinical tests for the gene in question are often not satisfactory. Thus, there is a need to develop more accurate methods for diagnosis and prediction of prognosis of LUAC patients.

## Materials and Methods

### Sources of Research Data

The GSE68465 (Shedden et al., 2008), GSE42127 (Hight et al., 2020), and GSE30219 (Rousseaux et al., 2013) data sets (containing data from 443, 133, and 148 LUAC patients, respectively) were downloaded from the Gene Expression Omnibus database (Edgar et al., 2002). GSE68465 was defined as an internal group, and the other data sets were combined as an external group (test group). The batch effect was balanced using the “SVA” package in R (version 4.0.2) (Irizarry et al., 2003). When more than one probe was mapped to one gene ID, we took their average value for further analysis. In addition, mRNA expression profiles and corresponding clinical information of LUAC patients were obtained from The Cancer Genome Atlas (TCGA) database (Chang et al., 2015).

### Identification of LUAC-Specific Cell-Cycle-Related Genes (CCRGs)

Single-sample gene set enrichment analysis (ssGSEA) was implemented using the “GSVA” R package (Barbie et al., 2009). The reference gene sets were from the MSigDB2 database (Liberzon et al., 2011): “KEGG_CELL_CYCLE” (Kanehisa, 2002) and “GO_CELL_CYCLE” (Mi et al., 2019). Spearman correlations were calculated between pairs of enrichment scores and each gene. Next, genes that met both of the following criteria were defined as LUAC-related CCRGs: absolute correlation > 0.3 and *P* < 0.01. The analysis of differentially expressed genes used the “limma” package. | Log_2_ fold change| > 1 and *P* < 0.05 were the criteria for determining differentially expressed genes. Finally, LUAC-specific CCRGs were screened out.

### Construction and Verification of Prognostic Model

The samples from GSE68465 were divided into two groups at a ratio of 1:1 at random to form an internal training group and a test group. Univariate Cox regression analysis, LASSO regression, and multiple Cox regression analysis were used to investigate the prognostic value of CCRGs in predicting the overall survival (OS) of LUAC patients, and to construct a model. On the basis of the median value of the risk score, all samples in the training group were divided into low- and high-risk groups. Kaplan–Meier survival curves and time-dependent receiver operating characteristic curves for OS evaluation of the two groups were plotted to evaluate the accuracy of the signature in the internal training group. The results were further confirmed in the internal and external test groups. To demonstrate that the model represents an independent risk factor, the combination of the signature and clinical factors was further validated through univariate and multivariate Cox regression analysis. *P* < 0.05 was regarded as statistically significant.

### Establishment of Prognostic Prediction Model Line Graph

To predict 1, 3, and 5 years OS, we established a nomogram and plotted its calibration curve based on all independent prognostic factors determined by multivariate analysis. Cox regression analysis was conducted with the R software to observe the relationship between the predicted probabilities and the actual occurrence rates.

### Identification and Survival Rate Analysis of Subtypes

With the R package “ConsensusClusterPlus,” the gene expression matrix contained in the model was used to identify molecular subtypes in LUAC. Then, survival rate analysis was performed and displayed for single subtypes.

### Verification of Prognosis-Related CCRG Expression

Data from the Clinical Proteomic Tumor Analysis Consortium (CTPAC) (Rudnick et al., 2016) and the Human Protein Atlas (HPA) (Pontén et al., 2008) were chosen to verify the protein expression of LUAC-specific CCRGs in tumor tissues and normal tissues, and to determine whether the expression differences were consistent with the previous mRNA results from TCGA. Differences were considered notable if *P* < 0.05.

### Immunohistochemistry

After obtaining the consent of 3 LUAC patients, tissue sections were obtained from the pathology department of our hospital. After blocking with endogenous peroxide and protein, the sections were then incubated with diluted specific anti-ECT2 or anti-BUB1B at 4°C overnight. The next day, the sections were incubated with the secondary antibody at 37°C for 1 h. The sections were stained with 3,3-diaminobenzidine solution for 3 min and counterstained with hematoxylin. The slices are finally observed and photographed under a microscope.

## Results

### Screening for LUAC-Specific CCRGs

**FIGURE 1:**
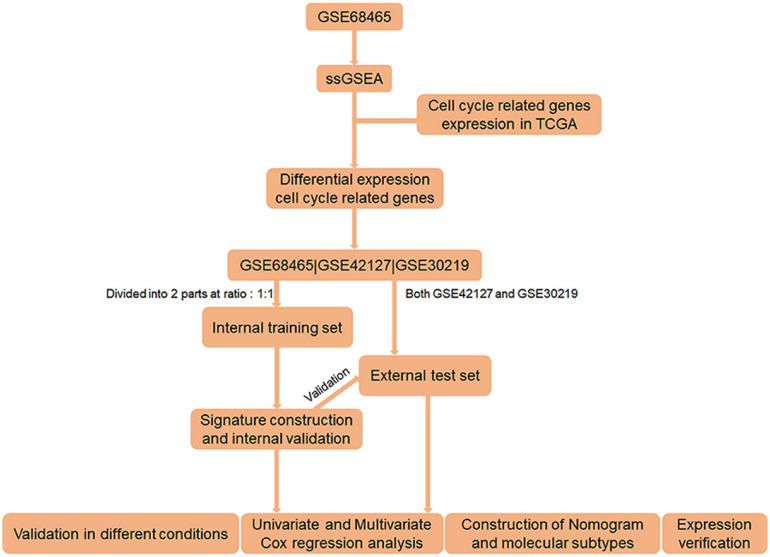
Flow chart of the entire research.

**FIGURE 2:**
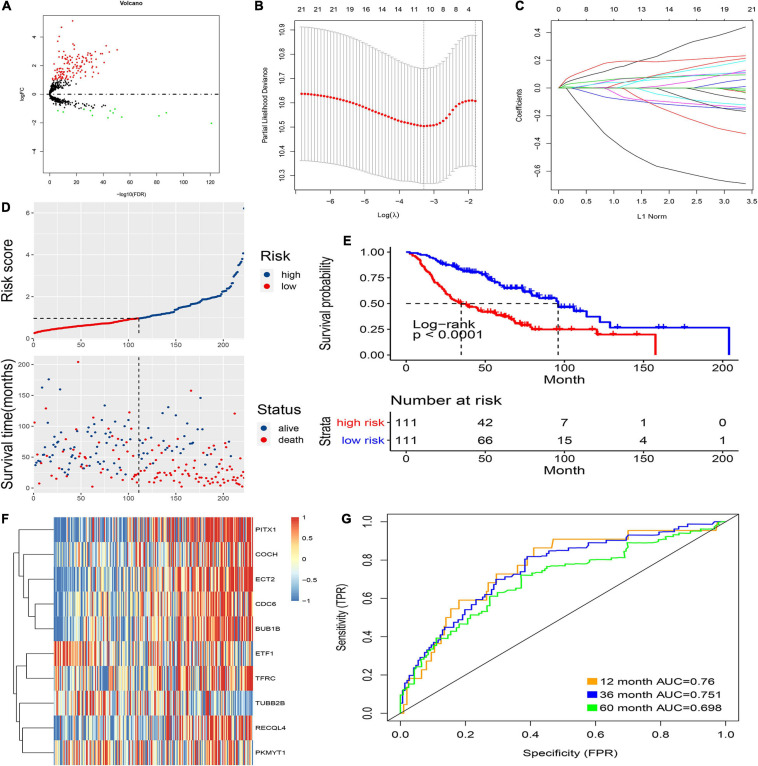
Prognostic model of the training cohort and risk signature with the 10 CCRGs. **(A)** LUAC-specific CCRGs were screened out. **(B)** The association between deviance and log(λ). **(C)** The association between coefficients of genes and log(λ). **(D)** Risk score of the high and low groups. **(E)** Survival analysis of the high and low groups. **(F)** Heatmap of the expression of 10 CCRGs. **(G)** The AUC of the ROC.

The workflow of the study is shown in Figure 1. On the basis of the mRNA data and clinical features from GSE68465, we conducted ssGSEA and used the CCRG sets as a reference, identifying 1,029 genes as LUAC-related CCRGs. Then, univariate Cox analysis was performed to screen out CCRGs that were significantly related to survival, resulting in 801 genes (Supplementary Table 1). By comparison with mRNA expression in the TCGA LUAC data set, we screened out 148 differentially expressed CCRGs (Supplementary Table 2) as LUAC-specific CCRGs (Figure 2A).

### Construction and Verification of Prognostic Model

First, the internal group (GSE68465) was randomly divided into two groups, which were used as an internal training group and a test group. Then, LASSO regression was used, and cross-validation was performed in the internal training group (Figure 2B). The preliminary signature included 21 CCRGs: ETF1, TFRC, RRM2, MYBL2, CDC6, RECQL4, BUB1B, ECT2, PITX1, TRIP13, RPL39L, PKMYT1, CKS1B, KIF23, MCM10, TUBB2B, COCH, DTL, CENPE, BLM, and DHRS2 (Figure 2C). Then, multivariate Cox regression was performed to build prognostic signatures on the basis of these CCRGs. Finally, a signature of 10 CCRGs was selected, and risk scores were calculated as follows: risk score = (−0.655 ^∗^ exp of ETF1) + (0.106 ^∗^ exp of TFRC) + (0.122 ^∗^ exp of CDC6) + (0.099 ^∗^ exp of RECQL4) + (0.178 ^∗^ exp of BUB1B) + (0.22 ^∗^ exp of ECT2) + (0.094 ^∗^ exp of PITX1) + (−0.212 ^∗^ exp of PKMYT1) + (−0.119 ^∗^ exp of TUBB2B) + (0.042 ^∗^ exp of COCH). After calculating the risk scores of individual patients, 1.0145 was chosen as the cutoff value to distinguish the high- and low-risk groups (Figure 2D). The survival analysis showed striking differences between the two groups (Figure 2E). The mRNA expression of the 10 LUAC-specific CCRGs in each sample is shown in Figure 2F. The accuracy was evaluated by the area under the curve (AUC) of the ROC curve; as shown in Figure 2G, the AUC values were 0.76 at 1 year, 0.751 at 3 years, and 0.698 at 5 years.

**FIGURE 3:**
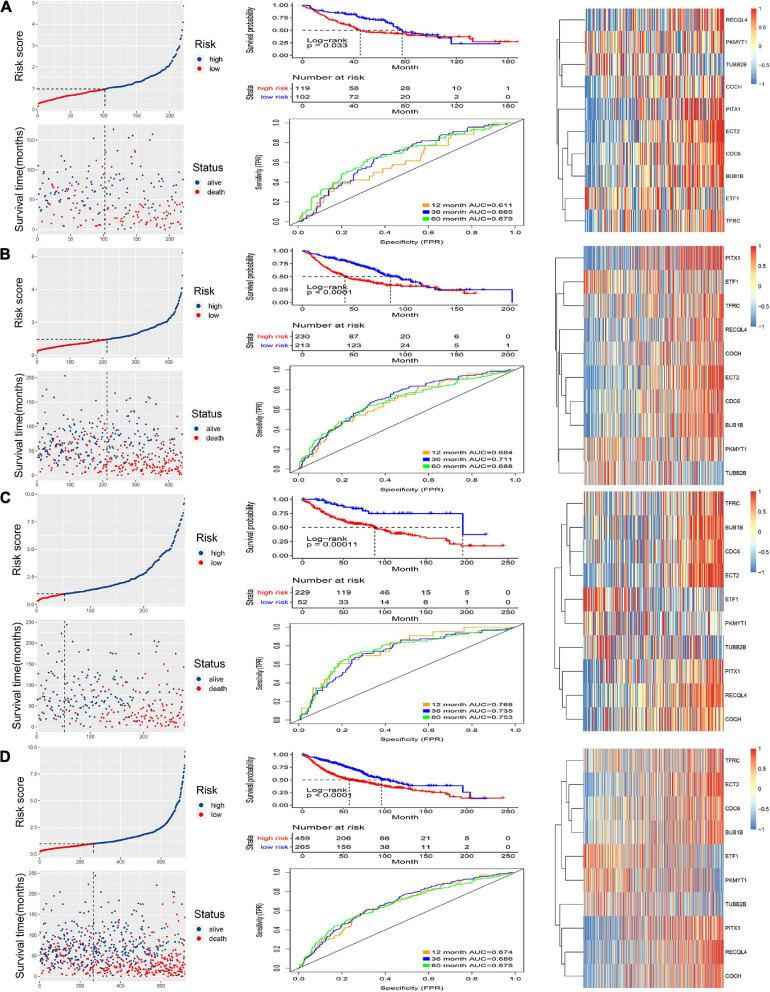
Validation of the signature. Risk score and survival analysis of the high and low groups, heatmap of the expression of 10 CCRGs, and the AUC of the ROC in **(A)** internal test group, **(B)** internal group, **(C)** external test group and **(D)** all samples.

The internal and external test groups were used to verify the accuracy of the model. Risk score analysis, survival analysis, and ROC analysis were performed repeatedly for each group (Figures 3A–D). The model could distinguish the high-risk group from the low-risk group efficiently. Survival analysis proved that the critical value remained valid. The AUC of the ROC curve in different groups further proved the robustness of the signature. The AUC values at 1, 3, and 5 years were 0.611, 0.665, and 0.679, respectively, for the internal test group; 0.664, 0.711, and 0.688 for the entire internal group; 0.768, 0.735, and 0.753 for the external validation group; and, finally, 0.674, 0.686, and 0.675 for all samples.

### Risk Score Is an Independent Prognostic Indicator of LUAC

**FIGURE 4:**
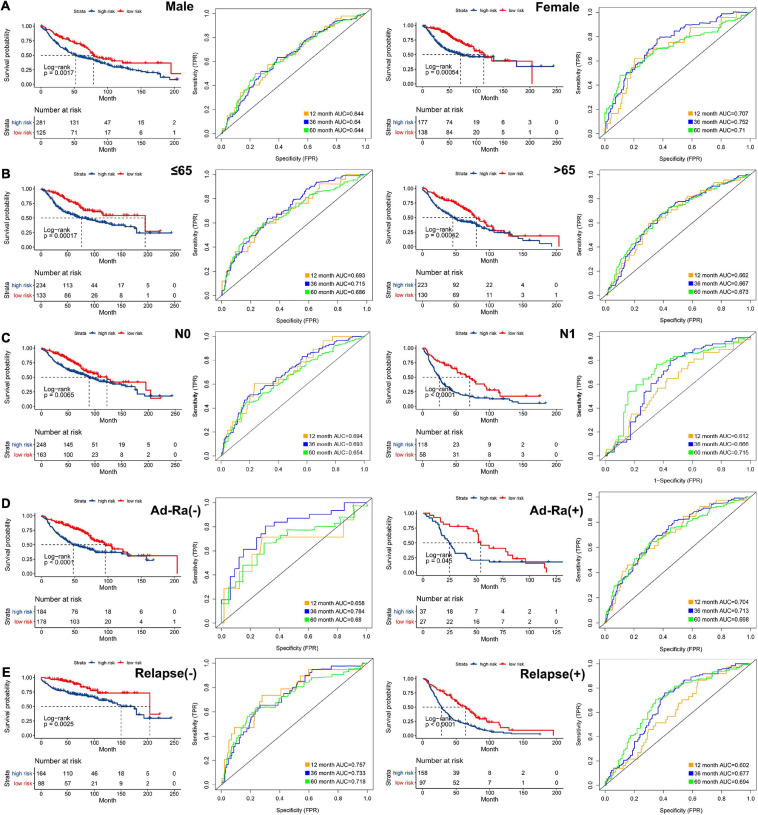
The AUC of the ROC that was computed by the signature under diverse situations. Survival analysis of the high and low groups and the AUC of the ROC in **(A)** gender, **(B)** age, **(C)** status of node metastasis, **(D)** whether they received adjuvant radiotherapy, and **(E)** whether relapsed.

**FIGURE 5:**
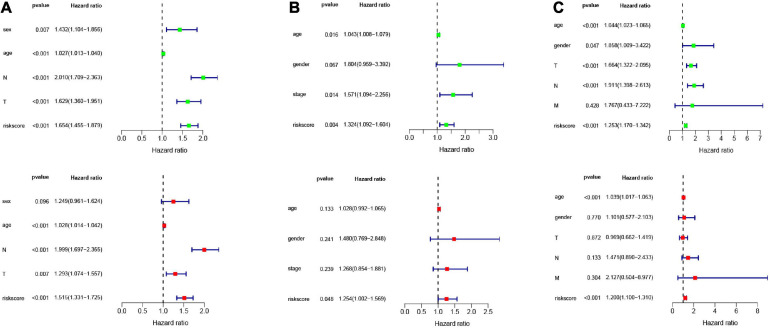
Univariate and multivariate Cox analysis of the signature combined clinical features. Univariate and multivariate Cox analysis in **(A)** GSE68465 **(B)** GSE42127 **(C)** GSE30219.

In order to analyze the efficiency of the signature in different situations, patients were divided into different groups on the basis of different clinical characteristics (age, gender, lymph node metastasis, whether they received adjuvant radiotherapy, and whether they relapsed). Figures 4A–E shows the results of ROC and survival analysis for each group under different conditions; the OS rate of the high-risk group was significantly lower than that of the low-risk group. These results showed that the signature was highly efficient and stable in different situations. In addition, we used univariate and multivariate Cox regression in GSE68465, GSE42127, and GSE30219 to analyze the prognostic value of the risk score in specimens with different clinicopathological factors (Figures 5A–C).

### Construction of the Nomogram Model

**FIGURE 6:**
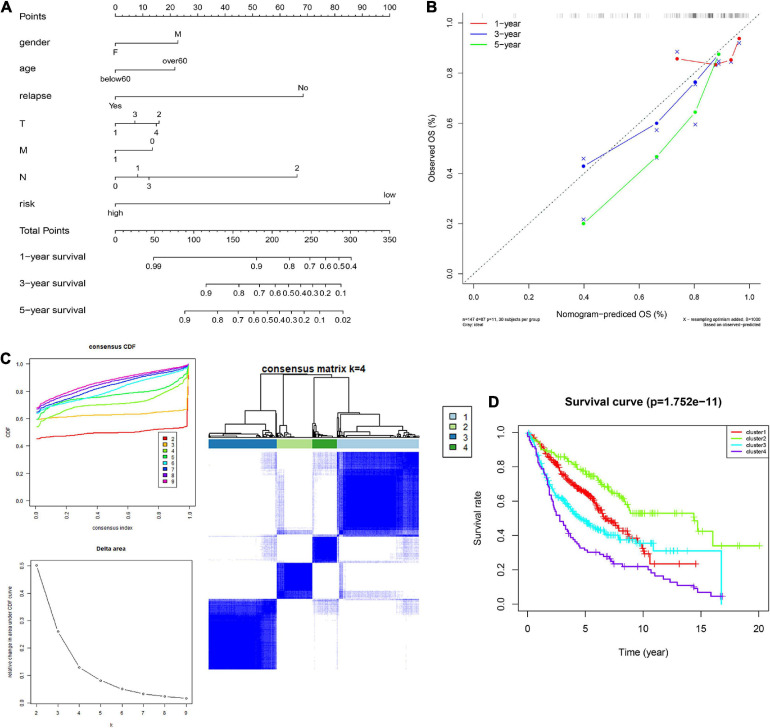
Construction and validation of the nomogram. **(A)** Details of the nomogram, **(B)** Calibration analysis based on the nomogram. **(C)** Molecular subgrouping based on 10 CCRGs: Elbow and gap plot for different numbers of subgroups; Consensus heatmap of the clusters. **(D)** Survival analysis of the four subgroups.

In order to integrate multiple predictors, show the relationship between the variables in the predictive model, we used the gender, age, relapse, T, M, N, and risk score to build a nomogram model (Figure 6A). The calibration curve was close to the ideal curve, indicating that the signature produced results consistent with the actual results (Figure 6B).

### Identification of Molecular Subtypes

We further verified whether the 10 CCRGs could divide patients into different molecular subgroups. The subgroup effect was most significant with a *k*-value of 4. OS analysis of different molecular subtypes confirmed the prognostic significance of molecular subtype classification methods for clinical patients (Figures 6C,D). These findings suggest that the 10 CCRGs are potential LUAC biomarkers that could have a vital role in clinical treatment.

### Protein Verification of Prognostic Genes

**FIGURE 7:**
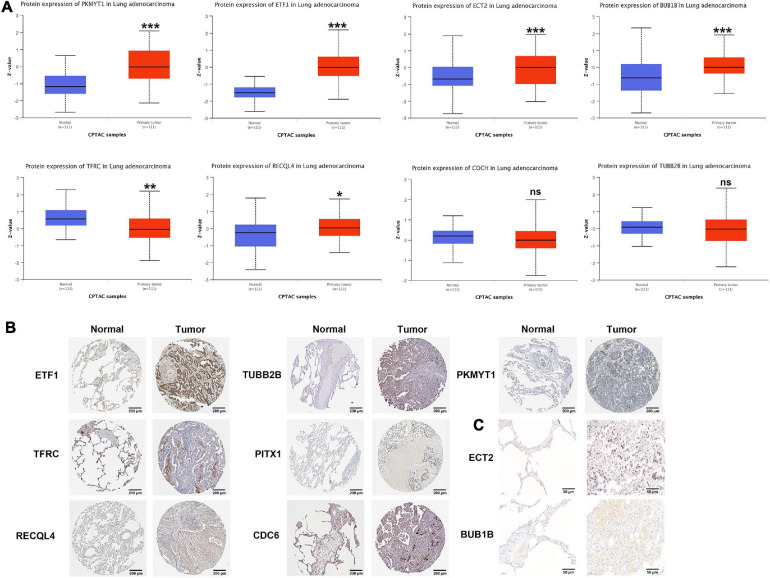
Protein level of CCRGs in LUAC tumor tissues and normal tissues. **(A)** CPTAC database. **(B)** HPA database. **(C)** IHC *P < 0.05, **P < 0.01, ***P < 0.001.

According to the CPTAC data, the protein expression of PKMYT1, ETF1, ECT2, BUB1B, and RECQL4 in tumor tissues was significantly increased, while the expression of TFRC was significantly reduced, and the expression of COCH and TUBB2B did not change significantly (PITX1 and CDC6 were not included) (Figure 7A). In the HPA data, compared with normal tissues, the expression of PKMYT1, ETF1, RECQL4, TUBB2B, and CDC6 in tumor tissues was remarkably upregulated, while the expression of TFRC and PITX1 was significantly downregulated (,ECT2, BUBB1B, and COCH were not included) (Figure 7B). In addition, through IHC, we found that ECT2 and BUBB1B are highly expressed in LUAC tissues (Figure 7C). The verification results basically coincided with the previous results.

## Discussion

LC is the most common type of cancer and a major contributor to cancer-related deaths worldwide. It accounts for 11.6% of all cancers, and there were approximately 2.1 million new cases in 2018 (Bray et al., 2018). In addition to the prevalence of LC, the prognosis of most LC patients is very poor, with a 5 years predicted survival rate of about 17.8% (Zappa and Mousa, 2016). LUAC is the main type of LC. Therefore, there is an urgent need to find a more accurate way to diagnose LUAC patients and predict their prognosis.

Recent research has confirmed that genes changes can regulate the cell cycle in cancer. Precise regulation of the cell cycle is a basic requirement for eukaryotic cell homeostasis. The progression of the cell cycle consists of five consecutive phases: G0, G1, S, G2, and M (Schafer, 1998). A complex balance of different cyclin-dependent kinases (CDKs) and cyclins determines whether a cell enters the G1 phase of the cell cycle (Wood and Endicott, 2018). In addition, the G1 and G2 cell cycle checkpoints are considered to be an important part of regulating the cell cycle and are regulated by a variety of molecules (Afshari and Barrett, 1993). An extensive regulatory network composed of CCRGs is indispensable for the progression of the cell cycle. Understanding the expression levels of these factors and their combined regulatory modes is essential to predicting patient outcomes and prognosis. The development of drugs targeting one or more CCRGs may be the general trend of LUAC treatment in the future. At present, there is no research specifically analyzing which genes in CCRGs have an impact on the prognosis of LUAC patients. Therefore, it is of great significance to screen out these genes that play an important role in the progression of LUAC and the prognosis of patients.

In this work, we aimed to develop a prognostic signature related to the cell cycle. LUAC samples were divided into an internal training group, internal test group, and external test group. We used the internal training group to establish a prognostic model through Cox and LASSO regression analysis, and used the internal test group and external test group to verify the model. PKMYT1, ETF1, ECT2, BUB1B, RECQL4, TFRC, COCH, TUBB2B, PITX1, and CDC6 were screened out. We also studied some clinical features that may affect the survival of LUAC patients and constructed a nomogram to prove that the model can be better translated into clinical applications. Although new CCRGs are discovered every day, the use of genetic signatures can highlight the most vital markers for clinical applications.

In addition, we used the expression of these 10 CCRGs to classify LUAC patients into four different molecular subtypes. These four groups showed differences in prognosis, suggesting that these 10 genes have potential applications in LUAC diagnosis and treatment. The CPTAC and HPA databases were used to verify the differential expression of these genes. PKMYT1 is a membrane-associated kinase that can negatively regulate the G2/M transition of the cell cycle by phosphorylation and inactivation of CDK1 (Schmidt et al., 2017). In the cytoplasm, PKMYT1 can also promote the cytoplasmic separation of CDK1, thereby promoting the activation of mitosis-promoting factor and accelerating the cycle process (Lolli and Johnson, 2005). ETF1 is dysregulated in various types of cancer (Dubourg et al., 2002). ECT2 is a necessary link between the cell cycle machinery and Rho signaling pathways involved in the regulation of cell division, and its exchange function relies on its phosphorylation during the G2 and M phases (Tatsumoto et al., 1999). Expression of the BUB1B gene cannot be detected in G1 but reaches a peak in G2/M, and its absence can cause genome instability and the progression of LC (Myslinski et al., 2007). RecQL4 can protect chromosome stability by coordinating and regulating cell proliferation and cell cycle progression (Fang et al., 2018). TFRC accelerates cell proliferation and metastasis by upregulating AXIN2 in epithelial ovarian cancer (Huang et al., 2020). Methylation levels of COCH are elevated in the plasma of NSCLC patients with lymph node metastasis (Chen et al., 2020). Upregulation of TUBB2B may contribute to the development of neuroblastoma (Liu and Li, 2019). The p53 gene is the direct transcriptional target of PITX1 (Liu and Lobie, 2007). The synergistic effects of CDC6 and cyclin E induce DNA replication in resting cells when CDC6 and CDT1 are ectopically expressed (Borlado and Méndez, 2008). Our signature was constructed and verified in a comprehensive cohort and could be used in clinical practice with a large number of genes.

However, our research had some limitations. Our results based on data from TCGA, CPTAC, and HPA partially proved that 10 CCRGs are dysregulated in LUAC, suggesting that they may have vital roles in the occurrence and development of LUAC. Functional experiments are needed to further uncover the possible molecular regulatory mechanisms of these CCRGs.

## Conclusion

The current study shows that the cell cycle pathway is mainly responsible for the occurrence and development of LUAC. A prognostic signature was constructed based on LUAC-specific CCRGs, which could be used for prognostic evaluation of LUAC patients. In addition, the specific CCRGs screened out could be used as new targets for the treatment of LUAC.

## Data Availability Statement

The datasets generated for this study can be found in the online repositories. The names of the repository/repositories and accession number(s) can be found in the article/Supplementary Material.

## Author Contributions

YF: research design. WJ and JX: experiment implementation and drafting the manuscript. GL: literature search. ZL: help modify articles and collate references. CZ: review and revision of the manuscript. All authors contributed to the article and approved the submitted version.

## Conflict of Interest

The authors declare that the research was conducted in the absence of any commercial or financial relationships that could be construed as a potential conflict of interest.

## Supplementary Material

Supplementary Table 1 Details of 801 CCRGs in LUAC.

Supplementary Table 2 Details of 148 LUAC-specific CCRGs in LUAC.

## Abbreviations

CCRGs: cell-cycle-related genes
NSCLC: non-small-cell lung cancer
LC: lung cancer
LUAC: lung adenocarcinoma
LUSC: lung squamous cell carcinoma
ssGSEA: single-sample gene set enrichment analysis
LASSO: least absolute shrinkage and selection operator
PKMYT1: protein kinase, membrane associated tyrosine/threonine 1
ETF1: eukaryotic translation termination factor 1
ECT2: epithelial cell transforming 2
BUB1B: BUB1 mitotic checkpoint serine/threonine kinase B
RECQL4: RecQ like helicase 4
TFRC: transferrin receptor
COCH: cochlin
TUBB2B: tubulin beta 2B class IIb
PITX1: paired like homeodomain 1
CDC6: cell division cycle 6.
